# Effects of exercise interventions on brain-derived neurotrophic factor levels in overweight and obesity: A systematic review and meta-analysis

**DOI:** 10.1016/j.jesf.2024.04.001

**Published:** 2024-04-03

**Authors:** Wilson KC. Leung, Suk-yu Yau, Yijian Yang, Anthony WL. Kwok, Eliza ML. Wong, Jasmine KM. Cheung, Edward WC. Shum, Simon C. Lam, Lorna KP. Suen

**Affiliations:** a School of Nursing, Tung Wah College, 16/F, Ma Kam Chan Memorial Building, 31 Wylie Road, Kowloon, Hong Kong SAR, China; b School of Medical and Health Sciences, Tung Wah College, 10/F, Ma Kam Chan Memorial Building, 31 Wylie Road, Kowloon, Hong Kong SAR, China; c Department of Rehabilitation Sciences, Faculty of Health and Social Sciences, The Hong Kong Polytechnic University, Kowloon, Hong Kong SAR, China; d Department of Sports Science and Physical Education, Faculty of Education, The Chinese University of Hong Kong, New Territories, Hong Kong SAR, China

**Keywords:** Brain-derived neurotrophic factor, Diabetes, Obesity, Cognition, Aerobic training, Resistance training

## Abstract

**Background:**

*/Objective*. An explosion in global obesity epidemic poses threats to the healthcare system by provoking risks of many debilitating diseases, including cognitive dysfunction. Physical activity has been shown to alleviate the deleterious effects of obesity-associated cognitive deficits across the lifespan. Given the strong neuroprotective role of brain-derived neurotrophic factor (BDNF) and exercise training as a known modulator for its elevation, this systematic review sought to examine the strength of the association between exercise and BDNF levels in healthy people with overweight and obesity.

**Methods:**

Six electronic databases (PubMed, MEDLINE, EMBASE, Web of Science, Ovid Nursing Database, and SPORTDiscus) were searched from their inceptions through December 2022. The primary outcome of interest was BDNF levels. Interventional studies (randomized and quasi-experimental) with English full text available were included. Risk of bias of the included studies was assessed using the Physiotherapy Evidence Database Scale. Data were extracted for meta-analyses by random-effects models.

**Results:**

Thirteen studies (*n* = 750), of which 69.2% (9/13) had low risk of bias, were included. In the meta-analysis, exercise interventions had no significant effect on resting BDNF levels (standardized mean difference: −0.30, 95% CI -0.80 to 0.21, *P* = 0.25). Subgroup analyses also indicated no effects of age and types of control groups being compared on moderating the association.

**Conclusion:**

To further inform the role of BDNF in obesity-related cognitive functioning, rigorous studies with larger samples of participants and raw data available were imperatively deserved.

## 1 Introduction

Global prevalence of obesity and overweight has skyrocketed by >50% over the past few decades.^1^ Relationships between increased adiposity and cognitive deficits are apparently observed across the lifespan,^2^^,^^3^ yet intentional weight loss by surgical or behavioural (diet, exercise, or a combination of both) strategies could effectively alleviate obesity-associated cognitive impairments.^4^

Numerous studies have substantiated that exercise training can improve cognitive functions or delay cognitive decline. Endurance training can promote memory,^5^ alleviate hippocampal volume loss,^5^, ^6^, ^7^ and improve brain structure and activity.^6^^,^^7^ Balance exercises by simultaneously challenging both sensory (i.e., vestibular, visual, and somatosensory) and neuromuscular control mechanisms have been suggested to improve memory and spatial cognition in older adults at risk of falls.^8^ Amongst children with obesity and overweight, physical activity (PA) interventions versus usual practice revealed improvement in executive function by as high as 9%.^9^ Habitual PA can also improve global cognition and frontal function of older adults with obesity or overweight, independent of known cognition-related confounders (e.g., age, sex, body weight, educational achievement, etc.).^10^ Thus, gaining a better understanding of the underlying mechanisms regarding the effects of physical exercise on cognition could surely provide a more concrete evidence to support the notion and hence increase the treatment options for obesity-associated cognitive deficit.

Brain-derived neurotrophic factor (BDNF) is a key member of neurotrophin family that is highly expressed and widely distributed in the central nervous system, especially hippocampus and cerebral cortex. Its functions include survival and maintenance of the nervous system by circulating neurogenesis or neuronal repair, neuronal survival, synaptogenesis, and neuroplasticity of both central and peripheral nervous systems.^11^ At neurons, the neurotrophic effect of BDNF is elicited through binding with tyrosine receptor kinase B, which therefore orchestrates a multitude of intracellular pathways, including Ras/MAPK and PI3K/Akt cascades.^12^ While exercising, skeletal muscle contraction triggers BDNF synthesis in myocytes and its secretion into the bloodstream.^13^ Given that BDNF can cross the blood brain barrier and shuttle between the brain and the blood circulation,^14^ the peripheral levels of BDNF are considered a good representation of its cortical levels of brain.^15^^,^^16^ In Alzheimer's disease patients, reduced BDNF expression was apparently observed in hippocampus and cerebral (frontal, parietal, temporal) cortex.^17^ Therefore, drug-induced BDNF increments through alleviation of amyloid beta accumulation, synaptic dysfunction, and neuroinflammation is increasingly considered a valuable neuro-therapeutic option for the disease. Given that obesity, poor cognitive performance, and their interrelationships are strongly associated with low BDNF^18^, ^19^, ^20^ and exercise training could improve cognition via BDNF enhancement among people with overweight and obesity,^21^, ^22^, ^23^ it is strongly believed that the major contributor accounting for the cognitive benefits of exercise training in obesity could be linked to enhancement of BDNF expression.

A recent meta-analytic review revealed a significant effect size of BDNF increase following both acute and long-term exercise training.^24^ However, in type 2 diabetes (i.e., obesity is the leading cause of type 2 diabetes^25^), pooled mixed findings were observed.^26^ Since there is no systematic review examining the causal relationship between exercise training and BDNF production in people with overweight and obesity which usually precedes the onset of type 2 diabetes,^27^ this study was hence conducted to settle controversies arising from the two apparently conflicting systematic reviews. Besides, a better understanding of BDNF involvement in the context of obesity (i.e., a pre-disease stage) could shed light on the underpinning mechanisms of physical exercise to alleviate cognitive deficit along metabolic disease progression and hence a timely targeted preventive strategy can be proposed. Given that lower peripheral/brain levels of BDNF are largely implicated in the pathogenesis of many neurodegenerative disorders,^17^^,^^28^^,^^29^ the primary aim of this study was to systematically investigate the effects of varying exercise interventions on BDNF in people with overweight and obesity who are at risk of cognitive deficit. Since recent systematic reviews have shown that physical exercise can prevent cognitive impairment in obese subjects^30^^,^^31^ and there was a potential link between improved exercise-related cognitive outcomes and BDNF enhancement,^32^, ^33^, ^34^, ^35^ we hypothesized that exercise would increase BDNF levels in people with overweight and obesity.

## 2 Methods

### 2.1 Study design

The study protocol was developed in accordance with the Preferred Reporting Items for Systematic Reviews and Meta-Analyses (PRISMA) guidelines.^36^ Reporting of the study flow and findings was in line with the 2020 updated guideline for reporting systematic reviews.^37^ The protocol was registered in the PROSPERO registry (CRD42023414868), and published in *BMJ Open.**^38^*

### 2.2 Eligibility criteria

This review included interventional studies (RCTs and quasi-experimental studies), which examined the effects of exercise interventions on BDNF levels in healthy individuals with overweight or obesity.

### 2.3 Information sources

Potential studies were identified using six electronic databases (PubMed, MEDLINE, EMBASE, Web of Science, Ovid Nursing Database, and SPORTDiscus) from their inceptions through December 2022. Only studies with full text available and in English language were included. To avoid missing any eligible studies, the references of all included articles or searched review papers were also screened.

### 2.4 Search strategy

The Text Word terms used in the electronic database search (title/abstract/subject/keywords) were obese, obesity, overweight, metabolic syndrome, physical activity, exercise*, resistance training, aerobic training, functional training, exergame, exergaming, cognitive, cognition, BDNF, and brain-derived neurotrophic factor. The search queries for each database were summarized in Supplementary Material.

### 2.5 Types of participants

The present study included healthy human subjects with overweight or obesity. Obese or overweight participants having pathological conditions (e.g., type 2 diabetes) were excluded.

### 2.6 Types of interventions

Standalone or combined exercise interventions had to be included in at least one arm within the studies. Exercise interventions in combination with non-exercise interventions (e.g., diet control) in a multimodal program were excluded because the exercise effects on BDNF in obesity cannot be solely studied.

### 2.7 Types of comparison controls

Comparison groups across the included trials were categorized into either active or non-active controls. For active controls, we defined as exercise interventions at lower intensity or training dosages or behavioural strategies interrupting sedentary behavior. For non-active controls, we defined as non-exercise interventions, including “diet control”, “usual care”, “no treatment”, and “wait-list control”.

### 2.8 Outcome measures

The primary outcome of interest was BDNF levels (serum, plasma, whole blood, urine, etc.) in response to chronic or acute exercise interventions.

### 2.9 Study selection and data extraction

The searched articles were screened by the first authors (Leung WK and Yau SY) initially based on their titles and abstracts, followed by the full texts. The extracted information, including authors, publication year, number of participants in the intervention group and their characteristics, details of interventions and controls [e.g., training volume (frequency х intensity х time), program duration, and attrition/dropout], and key findings (i.e., changes in the BDNF levels), were summarized into an evidence table. All data were finally checked for relevancy by independent investigators (Lam SC and Suen LKP).

### 2.10 Methodological quality assessment

The methodological quality of the included studies was examined by the first authors (Leung WK and Yau SY) using the Physiotherapy Evidence Database (PEDro) scale. The PEDro scale is a reliable and valid instrument for assessing the methodological quality of RCTs and non-RCTs regarding the effects of exercise interventions on cognitive functioning.^39^^,^^40^ In brief, the PEDro scale consists of 11 items, where we were required to fill out “no” or “yes”. For each “no” or “yes” response, we assigned a value of 0 or 1, respectively. A total score for each study ranged from 0 to 11. As blinding (especially subjects and therapists) was not easily implemented in exercise intervention trials,^41^ the methodological quality classification of each article was adjusted with eligibility criteria considered as previously described [sum scores: ≥6 (“high quality, low risk of bias”); scores: 4–5 (“acceptable quality, moderate risk of bias”), and scores: ≤3 (“low quality, high risk of bias”)].^39^^,^^40^^,^^42^ The results were finally verified by independent investigators (Lam SC and Suen LKP).

### 2.11 Statistical analyses

For controlled trials, pairwise meta-analysis of post-intervention BDNF data [mean and standard deviation (SD)] between intervention and control groups was conducted using a random-effects model, which takes into account possible variations in effects sizes across trials.^43^ For continuous outcomes that were measured using different scales or the same unit of measures, data were summarized as standardized mean difference (SMD) or weighted mean difference (WMD), with 95% confidence interval (CI), respectively. The pooled estimates of effect size for each outcome were interpreted as small (0.2–0.49), medium (0.5–0.79), or large (≥0.8) according to the Cohen's rule of thumb for effect sizes.^44^ The degree of heterogeneity across studies was assessed using Higgins *I*^*2*^ statistics. Results of the *I*^*2*^ statistics in 0–25%, 25–50%, and >50% represented low, moderate, and high heterogeneity, respectively. In order to assess publication bias, funnel plots were constructed when there were at least 10 studies in the meta-analysis. In case of missing data, we contacted the authors and addressed the possible impacts of missing information on our synthesized evidence in the discussion. All meta-analyses were conducted using Review Manager (RevMan version 5.4) software.

## 3 Results

### 3.1 Study selection

**Fig. 1:**
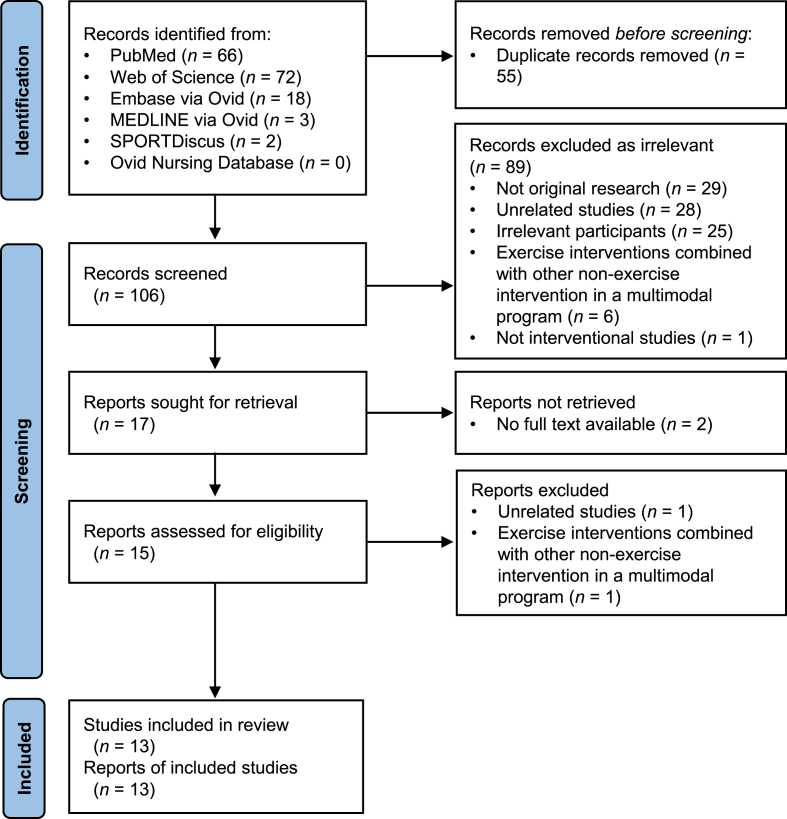
Study selection flow.

By December 2022, our electronic database search retrieved a total of 161 records. After removing the duplicates, we screened the titles and abstracts of 106 studies. Full texts of 15 potentially eligible studies were then retrieved for assessment. After the full text assessment, two trials were excluded due to unrelated research objectives (i.e., no neurocognitive measures^45^) and non-standalone exercise interventions [i.e., in combination with other weight reduction approaches (e.g., diet control)^46^]. Finally, 13 studies were included for review and four studies with raw data available were included for meta-analysis. Fig. 1 showed the study selection process.

### 3.2 Characteristics of included trials

Of the 13 included studies, eight (61.5%) were randomized trials^22^^,^^23^^,^^47^, ^48^, ^49^, ^50^, ^51^, ^52^ and five (38.5%) were quasi-experimental trials (nonrandomized or single-group pre-post trials).^21^^,^^53^, ^54^, ^55^, ^56^The interventional trials had one to four interventional and/or control arms. The 13 studies had a total of 750 participants, and the number of participants across studies varied from 6 to 304. The proportion of female sex ranged from 0% to 100%. Their body weight statuses were classified as overweight or obesity according to body mass index (BMI) (27.8 kg/m^2^ to 38.2 kg/m^2^) or percent body fat >30% for adults and older adults, as well as the World Obesity Federation cutoff points for children and adolescents. Sample types for BDNF measurement included serum,^21^, ^22^, ^23^^,^^47^^,^^48^^,^^50^^,^^52^, ^53^, ^54^, ^55^ plasma,^51^ and urine.^56^ Neurocognitive measures included overall cognitive/executive function,^23^^,^^48^^,^^53^ cognitive inhibition,^21^^,^^22^^,^^47^^,^^49^^,^^52^ working memory,^22^^,^^48^^,^^49^^,^^52^^,^^56^ sustained attention,^22^^,^^48^ mental flexibility,^49^^,^^52^ intelligence,^23^^,^^47^ processing speed,^48^^,^^49^ and brain/hippocampal structure or activity.^51^^,^^54^ Characteristics of the 13 included studies were summarized in Table 1.

**Table 1:** Characteristics of the included interventional trials published from 2015 to 2022 (*n* = 13).

	Number of study (%)
Study design	
Randomized trials	8 (61.5)
Quasi-experimental trials	5 (38.5)
Study participants	
Children and adolescents	2 (15.4)
Adults	9 (69.2)
Older adults	2 (15.4)
% women^a^	0–100%
Sample size^a^	6–304
Sample types for BDNF measurement	
Serum	10 (76.9)
Plasma	1 (7.7)
Urine	1 (7.7)
Not specified	1 (7.7)
Neurocognitive measures	
Overall cognitive/executive function	3 (23.1)
Cognitive inhibition	5 (38.5)
Working memory	5 (38.5)
Sustained attention	2 (15.4)
Mental flexibility	2 (15.4)
Intelligence	2 (15.4)
Processing speed	2 (15.4)
Brain structure or activity	2 (15.4)

BDNF, brain-derived neurotrophic factor.

a Data were presented as range.

### 3.3 Methodological quality assessment

Table 2 showed the details of methodological study assessment for each included study. The overall quality rating of all included studies was high, with a mean score (±SD) of 6.15 (±1.99); 69.2% (9/13) were rated as having high quality, 15.4% (2/13) as having acceptable quality, and 15.4% (2/13) as having low quality. All trials demonstrated clear eligibility criteria, while 84.6% (11/13) considered intention-to-treat analysis. Also, 84.6% (11/13) of the studies involved between-group statistical comparisons and provided both point measures as well as measures of variability for at least one key outcome. Of studies involving two or more arms, 90.9% (10/11) showed that the groups were similar at baseline about the body weight status (e.g., BMI, %body fat, etc.) and/or pre-training status (e.g., resting heart rate). However, none of the studies considered blinding of subjects or therapists. Blinding of outcome assessors was also only found in two studies.^48^^,^^50^

**Table 2:** Methodological quality assessment of included studies (*n* = 13).

Study	Eligibility criteria	Random allocation	Allocation concealment	Similar at baseline	Subject blinding	Therapist blinding	Assessor blinding	Dropout rate	Intention-to-treat analysis	Between-group comparisons	Point measures	Total scores	Overall quality
Alizadeh and Dehghanizade (2022)	1	0	1	1	0	0	0	0	1	1	0	5	Acceptable
de Lima et al. (2022)	1	1	1	1	0	0	0	0	1	1	1	7	High
Rodriguez-Ayllon et al. (2022)	1	1	1	1	0	0	0	0	0	1	1	6	High
Li et al. (2021)	1	1	1	1	0	0	0	1	1	1	1	8	High
Zlibinaite et al. (2021)	1	1	1	1	0	0	0	0	1	1	1	7	High
Bergman et al. (2020)	1	1	1	1	0	0	0	0	1	1	1	7	High
Inoue et al. (2020)	1	1	1	1	0	0	0	1	0	1	1	7	High
Kim and Kang (2020)	1	0	0	0	0	0	0	0	1	0	1	3	Low
Wheeler et al. (2020)	1	1	0	1	0	0	1	1	1	1	1	8	High
Goldfield et al. (2018)	1	1	1	1	0	0	1	1	1	1	1	9	High
Rodriguez et al. (2018)	1	0	1	0	0	0	0	0	1	1	1	5	Acceptable
Russo et al. (2017)	1	0	1	1	0	0	0	0	1	1	1	6	High
Mueller et al. (2015)	1	0	0	0	0	0	0	0	1	0	0	2	Low
1 = Yes, 0 = No													

For the meta-analysis, all four included studies^23^^,^^50^^,^^51^^,^^56^ were rated high quality, having a median score of 7 (range 6–9), which was higher than the median PEDro score (i.e., 4) for all studies falling in the discipline of sports physiology.^41^

### 3.4 Exercise interventions and BDNF levels

Table 3 summarized the findings of the 13 trials by three age groups. Two (15.4%), nine (69.2%), and two (15.4%) of them were conducted in children and adolescents (aged 8–18 years),^50^^,^^51^ in adults (aged 18–70 years),^21^^,^^22^^,^^47^^,^^49^^,^^52^, ^53^, ^54^, ^55^, ^56^ and in older adults (aged 60–73 years),^23^^,^^48^ respectively. For exercise interventions, three studies (23.1%) examined acute exercise effects,^48^^,^^55^^,^^56^ while 10 trials (76.9%) studied chronic interventions on resting BDNF lasting 6 weeks to 13 months varying from 2 to 5 sessions per week, with each lasting 20–90 min.^21^, ^22^, ^23^^,^^47^^,^^49^, ^50^, ^51^, ^52^, ^53^, ^54^ The adherence rates were 56–96%,^50^, ^51^, ^52^ and the attrition/dropout rates ranged from 0 to 15%.^22^^,^^23^^,^^48^, ^49^, ^50^, ^51^, ^52^^,^^54^

**Table 3:** Summary of interventional trials for examining effectiveness of exercise interventions on brain-derived neurotrophic factors and cognitive functioning in individuals with overweight and obesity (*n* = 13).

Authors (publication year); region; study design	Participants	Intervention	Control	Intensity	Duration	Training volume	Main findings^a^	Attrition rate^b^
Children and Adolescents								
Rodriguez-Ayllon et al. (2022); Spain; 2-arm randomized trial	Overweight/obese children	Aerobic and resistance exercises, supplemented with playful activities and games involving coordinative exercises	Usual care (wait-list)	>80% of HR_max_	20 weeks	90 min × 3–5 sessions/week	No effect on plasma levels of BDNF and other neurologic biomarkers.	12.5%
	- BMI according to the WOF cutoff points						No mediator effects of the biomarkers between exercise and cognitive function.	
	- Age: 8–11.9 years							
	- *n* = 81 (41% female)							
Goldfield et al. (2018); Canada; 4-arm randomized trial	Overweight/obese adolescents	1) Aerobic exercise (treadmill, elliptical machine, and/or cycle ergometer)	Non-active (diet counselling only)	1) Aerobic: 8%	22 weeks	20–90 min × 4 sessions/week	No effect on serum BDNF.	1) Aerobic: 65–85% HR_max_
	- BMI >95th percentile	2) Resistance exercise (weight machines or free weights)		2) Resistance: 8-RM				2) Resistance: 10.3%
	- Age: 14–18 years	3) Combined						3) Combined: 1.3%
	- *n* = 304 (70% female)							
Adults								
Alizadeh and Dehghanizade (2022); Iran; 3-arm nonrandomized trial	Obese women (active/inactive)	Functional training (resistance, circuit training)	Usual care	Borg scale: 6-7	8 weeks	60 min × 3 sessions/week	Increased serum BDNF.	Not specified
	- BMI: ≥30 kg/m^2^						Executive function (inhibition) improved.	
	- Age: 20–35 years							
	- *n* = 25 (100% female)							
de Lima et al. (2022); Brazil; 2-arm randomized trial	Overweight/obese, sedentary men	1) HIIT (sprinting)	No control	1) HIIT: 85–100% max velocity	8 weeks	60 min × 3 sessions/week	Increased serum BDNF.	0% for both groups
	- BMI: ≥25 kg/m^2^	2) MICT (running)		2) MICT: 60–75% HR_max_			Executive function (inhibition, and working memory) improved.	
	- Age: 30–50 years							
	- *n* = 25 (100% male)							
Zlibinaite et al. (2021); Lithuania; 2-arm randomized trial	Overweight/obese adults	Ergometer cycling	Usual care	50–60% VO_2max_	8 weeks	60 min × 5 sessions/week	No effect on serum BDNF levels, cognitive and motor functions.	0%
	- BMI: 33.5 ± 3.6 kg/m^2^						Body weight, VO_2_max, resting HR and BP improved.	
	- Age: 38–56 years						No effect on heart variability.	
	- *n* = 33 (100% female)							
Bergman et al. (2020); Sweden; 2-arm randomized trial	Overweight/obese office workers	Treadmill workstation plus encouraging emails	Sit-stand office desk	Not specified	13 months	60 min × 5 weekdays	No effect on blood BDNF levels.	15%
	- BMI: 29.3 ± 3.8 kg/m^2^						Increased weekday walking time.	
	- Age: 40–67 years						Positive associations between changes in walking time or LPA and hippocampal volume.	
	- *n* = 80 (55% female)						Negative associations between sitting time and hippocampal volume (adults aged 51 years and above).	
Inoue et al. (2020); Poland; 2-arm randomized trial	Obese men	Treadmill run:	No control	1) HIIT: 100% VO_2max_	6 weeks	40 min × 3 sessions/week	Acute and chronic effect on increased serum mature BDNF levels.	Not specified
	- BMI: 34.4 ± 3.5 kg/m^2^	1) HIIT		2) MICT: 65% VO_2max_			No acute or chronic effect on serum pro-BDNF levels.	
	- Age: 18–36 years	2) MICT					Executive function (inhibition) improved.	
	- *n* = 20 (100% male)						No effect on abdominal fat.	
Kim and Kang (2020); Korea; single-arm pre-post trial	Obese women (pre-/post-menopausal)	Resistance exercise (circuit training)	No control	55–65% 1RM	Not specified	60 min	Increased serum BDNF.	Not specified
	- %body fat: >30%						Increased serum level of other neuroplasticity factors (nerve growth factor and cathepsin B).	
	- Age: 40–69 years						Cognitive function improved.	
	- *n* = 52 (100% female)							
Rodriguez et al. (2018); United States; 2-arm nonrandomized trial	Obese men	HIIT (running)	MICT	80–90% VO_2max_	Single bout	30 min	Reduced %body fat.	Not specified
	- BMI: 38.2 ± 1.4 kg/m^2^						Increased serum BDNF.	
	- Age: 25.5 ± 1.7 years						Increased blood lactate.	
	- *n* = 6 (100% male)						No effect on plasma cortisol.	
Russo et al. (2017); Italy; 2-arm nonrandomized trial	Overweight/obese adults	Aerobic and resistance exercises (isoenergetic):	No control	1) 65% HRR	Single bout	Not specified	Reduced urinary BDNF.	Not specified
	- BMI: 35.4 ± 7.2 kg/m^2^	1) 65% HRR		2) 70% HRR			Larger reduction in urinary BDNF levels following exercise at a higher intensity (70% HRR).	
	- Age: 30–70 years	2) 70% HRR					Cognitive function (working memory) improved only after 65% HRR intensity training.	
	- *n* = 12 (67% female)							
Mueller et al. (2015); Germany; single-arm pre-post trial	Overweight/obese adults	Aerobic and resistance exercises	No control	70–80% HR_max_	3 months	60 min × 2 sessions/week	Increased serum BDNF levels in responders (63% participants).	0%
	- BMI: 33.6 ± 5.9 kg/m^2^						Reduced BMI, reduced serum leptin, and increased HDL.	
	- Age: 21–42 years						Associations of reduced leptin, increased HDL, and increased BDNF with increased grey matte density in the left hippocampus and altered diffusivity in directly neighboring white matter regions.	
	- *n* = 16 (56% female)						Positive correlation between exercise-associated changes in BDNF levels and grey matter density in left hippocampus, left incular cortex, and parts of left inferior cerebellum in BDNF responders.	
							Association between exercise-induced BMI reduction and increased grey matter density in the left cerebellum and right insular cortex.	
Older Adults								
Li et al. (2021); China; 3-arm randomized trial	Overweight/obese adults	Ergometer cycling:	Inactive	1) HIIT: 90% VO_2max_	12 weeks	65 min × 3 sessions/week	Increased levels of serum BDNF and other neurotrophic factors.	10% for both interventional groups
	- BMI: 27.8 ± 1.0 kg/m^2^	1) HIIT		2) VICT: 70% VO_2max_			No effect on cognitive function.	
	- Age: 60–73 years	2) VICT					Physical fitness (cardiorespiratory endurance, flexibility, balance, and reaction) improved.	
	- *n* = 29 (40% female)							
Wheeler et al. (2020); Australia; 3-arm randomized trial	Overweight/obese, sedentary older adults with normal cognitive function	Treadmill walking with or without subsequent walking breaks from sitting	Uninterrupted sitting (8 h)	1) Treadmill:	Single bout	30 min	Increased serum BDNF.	0%
	- BMI: 31.2 ± 4.1 kg/m^2^			−65–75% HR_max_			Working memory improved (in exercise group with breaks).	
	- Age: 67 ± 7 years			2) Walking breaks - RPE: 9-11			Executive function improved (in exercise group without breaks).	
	- *n* = 67 (52% female)			- RPE: 12-15			No association between BDNF and cognitive function.	

Abbreviations: BDNF, brain-derived neurotrophic factor; BMI, body mass index; BP, blood pressure; HDL, high-density lipoprotein; HIIT, high-intensity interval training; HR, heart rate; HR_max_, maximal heart rate; HRR, heart rate reserve; LPA, light physical activity; MICT, moderate-intensity continuous training; RM, repetition maximum; RPE, rate of perceived exertion; VICT, vigorous-intensity continuous training; VO_2max_, maximal oxygen consumption; WOF, World Obesity Federation.

a All main findings represented outcome measures with either within- or between-group differences from baseline to the completion of intervention.

b Only attrition rates for the intervention groups were reported.

*Children and adolescents.* Exercise had no within- or between-group effects on resting BDNF levels.^50^^,^^51^ There were also no mediator effects of the changes of resting BDNF between exercise and altered brain structure or function (hippocampal structure and function, cognitive performance, and mental health).^51^

*Adults.* Of the eight studies on blood examination, six (75%) demonstrated elevated serum/blood levels of BDNF after a single bout (exercise BDNF) or longer durations of exercise programming (resting BDNF).^21^^,^^22^^,^^47^^,^^49^^,^^52^, ^53^, ^54^, ^55^ Favorable cognitive outcomes with resting BDNF elevation included inhibition,^21^^,^^22^^,^^47^ working memory,^22^ overall cognitive function,^53^ and increased hippocampal volume or altered hippocampal mean diffusivity.^54^ However, urinary BDNF levels were significantly declined immediately following an acute bout of combined interventions (aerobic plus resistance training), and working memory was only improved with exercise training at 65% of heart rate reserve (HRR), but not 75% of HRR.^56^

*Older adults.* Aerobic exercises, including acute treadmill running and regular ergometer cycling, at moderate-to-vigorous exercise intensities [65–75% of HR_max_ or 70–90% of maximal oxygen consumption (VO_2max_)] raised serum levels of both exercise and resting BDNF, respectively.^23^^,^^48^ Although acute exercise effects on executive function (e.g., working memory) were apparently observed, there were no significant associations between BDNF levels and cognitive function.^48^ No chronic exercise effects on cognitive function were also found.^23^

*Exercise modes.* Two studies consistently showed that resistance training at moderate intensity [Borg scale rating of 6–7 or 55–65% of 1-repetition maximum (1RM)] increased serum resting BDNF levels.^21^^,^^53^ However, aerobic exercises (treadmill or ergometer) at a similar level of physical exertion (50–60% of VO_2max_) had no effect on blood/serum resting BDNF.^49^^,^^52^ These studies were concomitantly conducted in adult populations.

*Sex difference*. There was no obvious sex disparity in either resting or exercise BDNF response to physical training as exemplified by BDNF elevations concomitantly observed in studies involving only men^22^^,^^47^^,^^55^ or only women.^21^^,^^53^ However, one study involving only middle-aged women following an 8-week intervention of ergometer cycling at a relatively low intensity (50–60% VO_2max_) did not show any significant effects on resting BDNF.^52^

### 3.5 Meta-analysis of exercise effects on BDNF levels

**Fig. 2:**
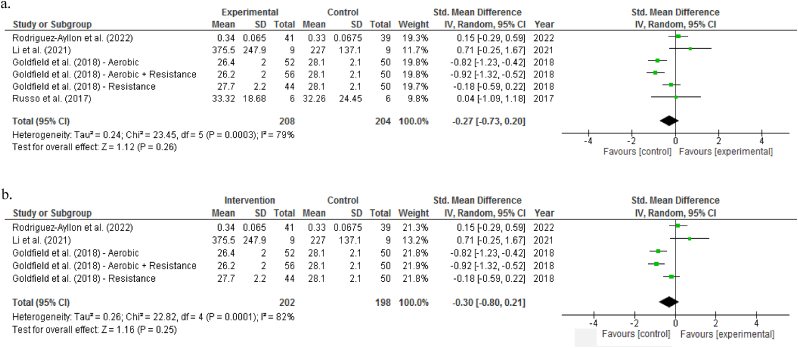
Forest plot showing overall effects of exercise interventions on (a) blood and urine levels of as well as (b) only blood levels of brain-derived neurotrophic factor.

Four trials that provided both point measures and measures of variability about BDNF levels were included for meta-analysis.^23^^,^^50^^,^^51^^,^^56^ Overall, the pooled analysis suggested no effects of exercise interventions on BDNF levels, with SMD -0.27 (95% CI -0.73 to 0.20, *P* = 0.26) and of high heterogeneity (*I*^*2*^ = 79%) (Fig. 2a). By only considering blood samples (i.e., excluding urine samples^56^) or chronic exercise effects (i.e., excluding acute bout of exercise training^56^), the pooled analysis still revealed no significant effects on resting BDNF (SMD -0.30; 95% CI -0.80 to 0.21; *P* = 0.25) (Fig. 2b).

**Fig. 3:**
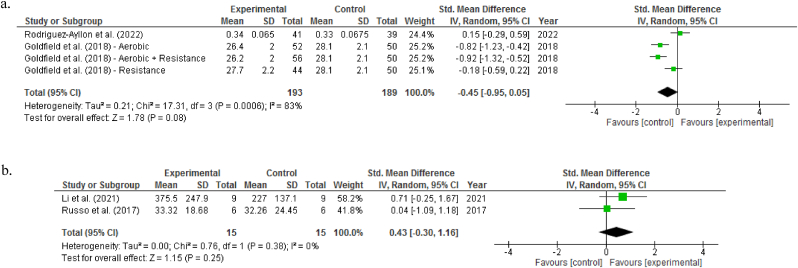
Forest plot showing effects of exercise interventions on brain-derived neurotrophic factor levels among children and adolescents (a) as well as adults and older adults (b).

Categorized by age groups, the subgroup/moderator analysis did not suggest any exercise effects on BDNF levels for both children and adolescents (SMD -0.45, 95% CI -0.95 to 0.05, *P* = 0.08)^50^^,^^51^ and adults and older adults (SMD 0.43, 95% CI -0.30 to 1.16, *P* = 0.25),^23^^,^^56^ with high (*I*^*2*^ = 83%) and low (*I*^*2*^ = 0%) heterogeneity, respectively (Fig. 3).

**Fig. 4:**
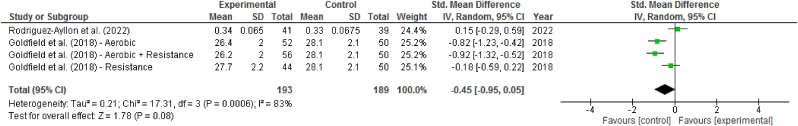
Forest plot showing effects of exercise interventions versus non-active controls on brain-derived neurotrophic factor levels.

When the sensitivity analysis was carried out by excluding the studies involving active controls, the pooled results also revealed no significant effect of exercise interventions on resting BDNF levels (SMD -0.45, 95% CI -0.95 to 0.05, *P* = 0.08), with considerable heterogeneity (*I*^*2*^ = 83%) (Fig. 4).^50^^,^^51^

Since none of the meta-analyses included 10 or more studies, we cannot assess for publication bias. Also, no further sensitivity analysis was carried out as all the included studies for the meta-analysis were of low risk of bias.

## 4 Discussion

To our knowledge, the present review was the first to evaluate the effects of exercise interventions on BDNF changes in healthy individuals with overweight and obesity. Our findings suggested no exercise effects on BDNF levels and the association was neither moderated by age nor types of control group. However, the insignificant results in our meta-analysis may be caused by insufficient statistical power to detect a significant difference. Future rigorous large-scale studies with raw data available are imperatively needed to examine the associations between exercise and BDNF expression in the context of obesity.

Given BDNF as a known contraction-induced myokine, it was reasonably believed that muscle strengthening could sensitize muscle to induce BDNF production.^57^ Our findings showed that resistance training conferred BDNF elevation on both young^21^ and middle-aged or older obese women,^53^ yet Szuhany et al. (2015) suggested no significant effects of resistance training on BDNF.^24^ One explanation for the heterogeneity was that the resistance training effects were sex-specific, leading to transient testosterone increments in women (total testosterone by 25%, free testosterone by 25%, and sex-hormone binding globulin by 4%),^58^ which in turn elicited BDNF increments in the female brain.^59^

Increased metabolic stress beyond thresholds to trigger adaptation during physical training was equally important for both men and women with overweight or obesity. Obese or overweight men exhibited BDNF elevation following both acute and chronic aerobic exercises at strenuous levels (>85% HR_max_^22^^,^^47^ or >80% VO_2max_^55^), while obese women similarly elicited obvious BDNF responses to resistance training at moderate intensities (Borg scale: 6–7 out of 10 ^21^ or 55–65% 1RM ^53^). However, there were no chronic endurance training effects at a relatively low intensity (50–60% VO_2max_) on BDNF in obese/overweight wom en.^52^ Future studies with rigorous study design should be guaranteed to verify the gender roles in BDNF responses to exercise interventions in obesity.

Strength of the present study was rigorous methodological design that was in line with the best reporting guidelines and based on a pre-specified protocol. Nonetheless, there were several study limitations. First, our meta-analysis had limited power to examine the exercise effects on BDNF due to a small number of eligible studies included. The insufficient power would lead to a lack of statistical significance in meta-analysis and multiple subgroup comparisons. The issue about the limited power in the meta-analysis due to limited sample size was consistently addressed in two other relevant systematic reviews.^24^^,^^26^ Second, missing raw data were found in many studies which only had graphical representation of data, thereby hindering a comprehensive meta-analysis. Therefore, meta-analytic comparisons between acute and chronic effects on BDNF were not possibly conducted because there was only one study providing raw dataset about the acute effects.^56^ Third, a number of different neurocognitive tasks were employed across studies, resulting in a great challenge in synthesizing evidence about the relationships between BDNF and cognitive outcomes. Other potential biases included mixed study designs, variability in population ages, lack of diversity and representation among study populations, and the omission of medical history information.

## 5 Conclusion

We observed that exercise conferred no effects on BDNF in overweight/obese individuals regardless of age and control groups. Although most trials were of high quality, this study was limited by an unavailability of raw data and methodological heterogeneity across the included studies.

## Funding

Collage Research Grant of the Tung Wah College (CRG2022/04)

***Protocol registration number.*** PROSPERO CRD42023414868.

## Funding/support statement

This work was supported by the Tung Wah College [grant number CRG2022/04]; Collage Research Grant, Hong Kong SAR, China.

## Declaration of Competing interest

The authors have no conflicts of interest relevant to this article.
