# Supplementary material for: Effects of exercise interventions on brain-derived neurotrophic factor levels in overweight and obesity: A systematic review and meta-analysis
Source: J Exerc Sci Fit. 2024 Apr 3;22(4):278–87. doi: 10.1016/j.jesf.2024.04.001 (PMC11015502; doi:10.1016/j.jesf.2024.04.001)
Supplement: Multimedia component 3 [file mmc3.docx]

**Supplementary Material**

1. **PubMed Search Strategy**

(("obes*"[All Fields] OR ("overweight"[MeSH Terms] OR "overweight"[All Fields] OR "overweighted"[All Fields] OR "overweightness"[All Fields] OR "overweights"[All Fields]) OR "metabolic syndrome"[All Fields]) AND ("physical activity"[All Fields] OR "exercis*"[All Fields] OR "resistance training"[All Fields] OR "aerobic training"[All Fields] OR "functional training"[All Fields] OR "exergam*"[All Fields]) AND "cogniti*"[All Fields] AND ("brain-derived neurotrophic factor"[MeSH Terms] OR ("brain derived"[All Fields] AND "neurotrophic"[All Fields] AND "factor"[All Fields]) OR "brain-derived neurotrophic factor"[All Fields] OR "bdnf"[All Fields] OR "brain-derived neurotrophic factor"[All Fields] OR "brain-derived neurotrophic factor"[All Fields])) AND ((fft[Filter]) AND (english[Filter]))

**Limiters**

- Language: English
- Text availability: Full text

1. **Web of Science Search Strategy**

All Fields = obes* OR overweight OR "metabolic syndrome"

AND

All Fields = "physical activity" OR exercis* OR “resistance training” OR “aerobic training” OR “functional training” OR exergam*

AND

All Fields = cogniti*

AND

All Fields = BDNF OR “brain-derived neurotrophic factor” OR “brain derived neurotrophic factor”

**Limiters**

- Document Types: Articles
- Language: English

1. **EMBASE Search Strategy via Ovid**

| 1. | exp obesity/ or obes*.mp. |
| --- | --- |
| 2. | overweight.mp. |
| 3. | metabolic syndrome.mp. |
| 4. | exp exercise/ or exercis*.mp. |
| 5. | physical activity.mp. |
| 6. | resistance training.mp. |
| 7. | aerobic training.mp. |
| 8. | functional training.mp. |
| 9. | exergam*.mp. |
| 10. | cogniti*.mp. |
| 11. | BDNF.mp. or exp brain derived neurotrophic factor |
| 12. | brain-derived neurotrophic factor.mp. or exp brain derived neurotrophic factor/ |
| 13. | 1 or 2 or 3 |
| 14. | 4 or 5 or 6 or 7 or 8 or 9 |
| 15. | 11 or 12 |
| 16. | 13 and 14 and 15 |
| 17. | 10 and 16 |
| 18. | limit 17 to (full text and english language) |

**Limiters**

- English Language
- Full Text

1. **Ovid Nursing Database Search Strategy via Ovid**

| 1. | obesity.mp. or exp Obesity/ |
| --- | --- |
| 2. | overweight.mp. |
| 3. | metabolic syndrome.mp. |
| 4. | exp Exercise/ or exercise.mp. |
| 5. | physical activity.mp. |
| 6. | resistance training.mp. |
| 7. | aerobic training.mp. |
| 8. | functional training.mp. |
| 9. | exergaming.mp. |
| 10 | exergame.mp. |
| 11. | cognitive.mp. |
| 12. | cognition.mp. |
| 13. | BDNF.mp. |
| 14. | brain-derived neurotrophic factor.mp. |
| 15. | Brain derived neurotrophic factor.mp. |
| 16. | 1 or 2 or 3 |
| 17. | 4 or 5 or 6 or 7 or 8 or 9 or 10 |
| 18. | 11 or 12 |
| 19. | 13 or 14 or 15 |
| 20. | 16 and 17 and 18 and 19 |
| 18. | limit 20 to (english language and full text) |

**Limiters**

- English Language
- Full Text

1. **MEDLINE Search Strategy via Ovid**

| 1. | Obes*.mp. or exp Obesity/ |
| --- | --- |
| 2. | overweight.mp. |
| 3. | metabolic syndrome.mp. |
| 4. | exp Exercise/ or exercise*.mp. |
| 5. | physical activity.mp. |
| 6. | resistance training.mp. |
| 7. | aerobic training.mp. |
| 8. | functional training.mp. |
| 9. | exp Exergaming/ or exergam*.mp. |
| 10. | cogniti*.mp. |
| 11. | BDNF.mp. or Brain-Derived Neurotrophic Factor/ |
| 12. | brain derived neurotrophic factor.mp. or Brain-Derived Neurotrophic Factor/ |
| 13. | 1 or 2 or 3 |
| 14. | 4 or 5 or 6 or 7 or 8 or 9 |
| 15. | 11 or 12 |
| 16. | 10 and 13 and 14 and 15 |
| 17. | limit 16 to (english language and full text) |

1. **SPORTDiscus Search Strategy**

( obes* OR overweight OR "metabolic syndrome" )

AND

( "physical activity" OR exercis* OR “resistance training” OR “aerobic training” OR “functional training” OR exergam* )

AND

cogniti*

AND

( BDNF OR “brain-derived neurotrophic factor” OR “brain derived neurotrophic factor” )

**Limiters**

- Language: English
- Full text
